# Effective Management of Acute Pulmonary Embolism and Deep Vein Thrombosis: Insights From a Case Series on Procedure Benefits

**DOI:** 10.7759/cureus.72694

**Published:** 2024-10-30

**Authors:** Salman Butt, Fazil Ashiq, Arun Kumar, Ragavendran Senniappan, Zeina Mashaal, Gopal Bhatnagar, Khurram Rasheed

**Affiliations:** 1 Heart, Vascular, and Thoracic Institute, Cleveland Clinic Abu Dhabi, Abu Dhabi, ARE; 2 Anesthesiology Department, Cleveland Clinic Abu Dhabi, Abu Dhabi, ARE; 3 Cardiothoracic Anesthesiology Department, Cleveland Clinic Abu Dhabi, Abu Dhabi, ARE

**Keywords:** acute pulmonary embolism, deep vein thrombosis, pulmonary circulation, pulmonary embolism, pulmonary thromboembolectomy

## Abstract

Pulmonary thromboembolectomy is an essential intervention for managing acute pulmonary embolism (PE), with various treatment approaches including systemic thrombolysis, open surgical embolectomy, and percutaneous mechanical thrombectomy. Multimodal approaches are crucial for improving outcomes in massive and submassive PE cases, with integration across disciplines such as vascular surgery and interventional radiology enhancing comprehensive care. This manuscript will discuss a case series featuring two patients diagnosed with acute PE. Both underwent successful percutaneous mechanical thrombectomy, demonstrating the procedure's effectiveness in improving outcomes across different patient profiles. These cases highlight the critical importance of early diagnosis and subsequent thrombectomy in treating acute PE.

## Introduction

Acute pulmonary embolism (PE) is a life-threatening condition characterized by the sudden blockage of the pulmonary arteries, typically caused by blood clots that travel to the lungs from the legs or other parts of the body. The standard treatment for PE involves anticoagulation, which helps prevent the formation of new clots while allowing the body's natural fibrinolytic mechanisms to break down the existing clot. However, anticoagulation alone may not be sufficient for all patients.

Pulmonary embolism can be stratified according to the European Society of Cardiology Guidelines as low, intermediate, or high risk. Approximately 30% of PE patients, classified as intermediate-high or high-risk, are better treated with procedural rapid removal of pulmonary artery clots and restore lung perfusion. Reperfusion treatments are critical in these high-risk scenarios to prevent hemodynamic instability and reduce the risk of mortality. These treatments include surgical pulmonary embolectomy or catheter-directed therapies.

Systemic thrombolysis involves the administration of thrombolytic drugs that dissolve blood clots throughout the circulation, not just in the lungs but also potentially causing bleeding elsewhere. Surgical pulmonary embolectomy, which physically removes the clot via an open surgical approach, is reserved for cases where thrombolysis is contraindicated or has failed and in patients who are hemodynamically unstable. Catheter-directed therapy offers a targeted approach where thrombolytic drugs are delivered directly within the pulmonary arteries versus mechanical thrombectomy entailing percutaneous devices designed to physically remove the clot.

Each of these treatments has specific indications and requires careful patient selection to balance efficacy with the risk of complications. This introduction to our discussion on pulmonary thromboembolectomy sets the stage for exploring how these advanced interventions can be strategically utilized to manage acute PE effectively, particularly through the detailed examination of specific case studies in our manuscript [1-5].

This report details a case series of two patients, a 72-year-old and a 32-year-old, who presented with different symptoms but were both diagnosed with acute PE. Despite their differing presentations and ages, both patients underwent successful mechanical thrombectomy performed by the vascular surgery team.

## Case presentation

Case 1

This case report outlines the clinical trajectory of a 72-year-old female patient with multiple comorbidities, including hypertension, hyperlipidemia, diabetes mellitus, and osteoporosis. The patient sustained a recent hip fracture, necessitating hip surgery two months ago at an external medical institution. Upon admission to our facility, she presented with palpitations and edema in her right lower limb, devoid of notable symptoms suggestive of respiratory distress, angina, or syncope. Notably, the patient had been receiving enoxaparin prophylaxis post-hip surgery.

**Figure 1:**
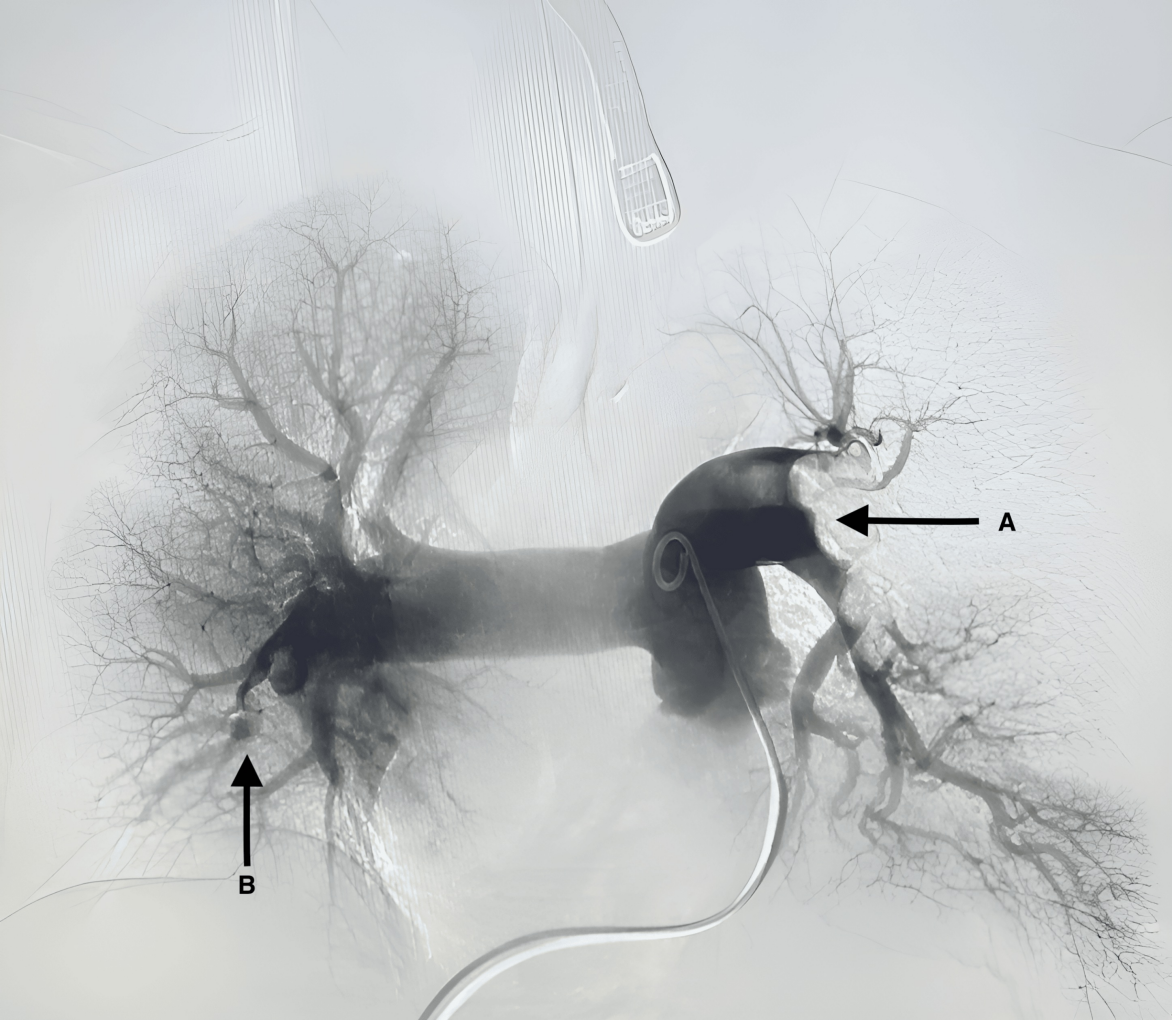
Pre-mechanical thrombectomy: submassive obstructive PE of the left pulmonary artery (A) and subsegmental PE of the right pulmonary artery (B) PE: pulmonary embolism

After being evaluated in the emergency department, the patient was diagnosed with acute submassive PE (Figure 1) and deep vein thrombosis (DVT) involving the proximal femoral, popliteal, and paired calf veins of her right lower limb. Angiography showed a submassive pulmonary embolism in the left main pulmonary artery, with flow absent on the left side and a near-occlusive clot in the basilar segment.

**Figure 2:**
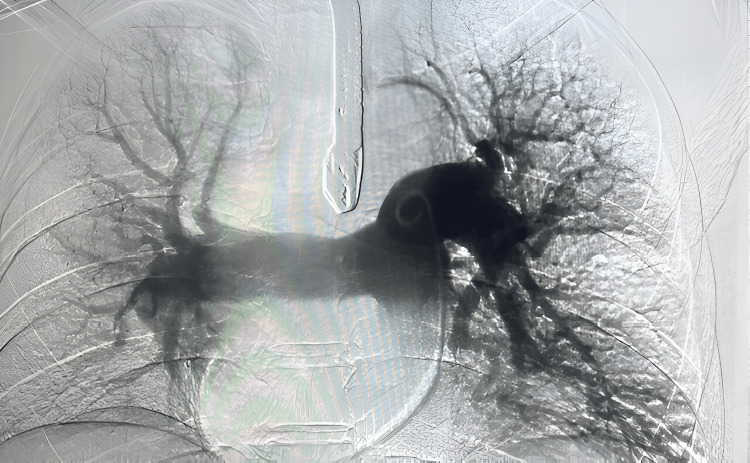
Post-pulmonary mechanical thromboembolectomy

An extra stiff Lunderquist wire (Cook Medical Inc., Bloomington, IN) was substituted, and the Penumbra Lightning Flash Indigo System CAT 16 device (Penumbra, Inc., Alameda, CA) was advanced to the level of the left pulmonary thrombus. Aspiration thrombectomy (Figure 2) was performed, extracting a substantial thrombus burden. Subsequent imaging displayed significant improvement, albeit with residual thrombus in the inferior and basilar segments, which were further addressed by additional aspiration thrombectomy.

After confirming satisfactory improvement through imaging with a pigtail catheter, 4 mg of tissue plasminogen activator (TPA) was administered within both the left and right pulmonary arteries.

**Figure 3:**
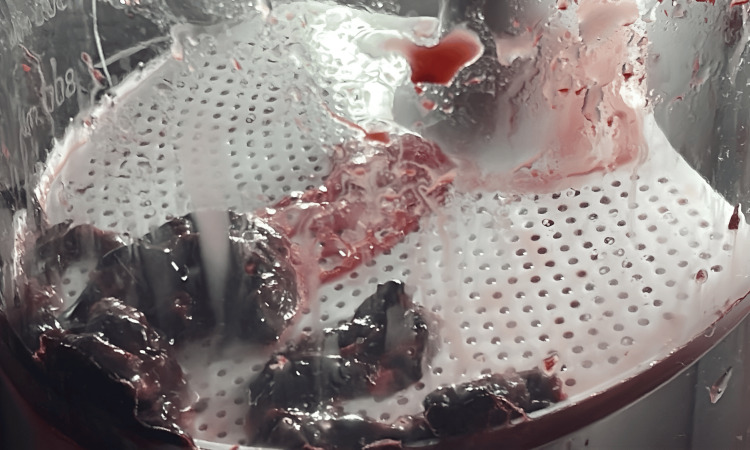
Thromboembolus within the Penumbra aspiration system

All wires, catheters, and the Penumbra device were withdrawn from the pulmonary arteries. The dry seal sheath was repositioned to the infrarenal inferior vena cava (IVC), where an angiogram was conducted, visualizing the renal veins for placement of a Bard Denali IVC filter device (Bard Peripheral Vascular, Inc., Tempe, AZ) (Figure 3).

Following the removal of all wires and sheaths, 25 mg of protamine was administered, and the previously placed Perclose device was deployed, with manual compression applied for hemostasis. General anesthesia was then discontinued, and the patient was transferred back to the ICU in stable condition. The patient was continued on anticoagulation postoperatively with anticoagulation for her DVT.

After being transferred from the ICU to the step-down unit, the patient was discharged home at her baseline functional status and continued to do well and remained asymptomatic when seen in the outpatient clinic two months post-discharge.

Case 2

A 32-year-old previously healthy male patient was admitted for shortness of breath and a recent episode of syncope, with no recent history of travel, trauma, or prolonged immobilization. On admission, vital signs were normal except for tachycardia. Respiratory physical examination was insignificant. ECG showed sinus tachycardia with mild ST depression in the lateral leads. Laboratory findings revealed high troponin and lactic acid. Based on the patient's presentation and investigations, a diagnosis of pulmonary embolism was suspected.

**Figure 4:**
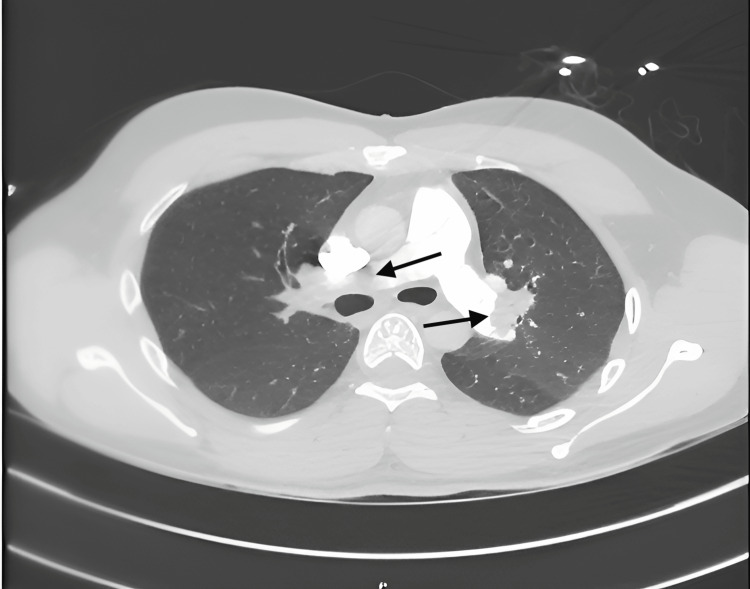
CTA demonstrating massive pulmonary embolism completely obstructing the left pulmonary artery and partially obliterating the right pulmonary artery CTA: computed tomography angiography

**Figure 5:**
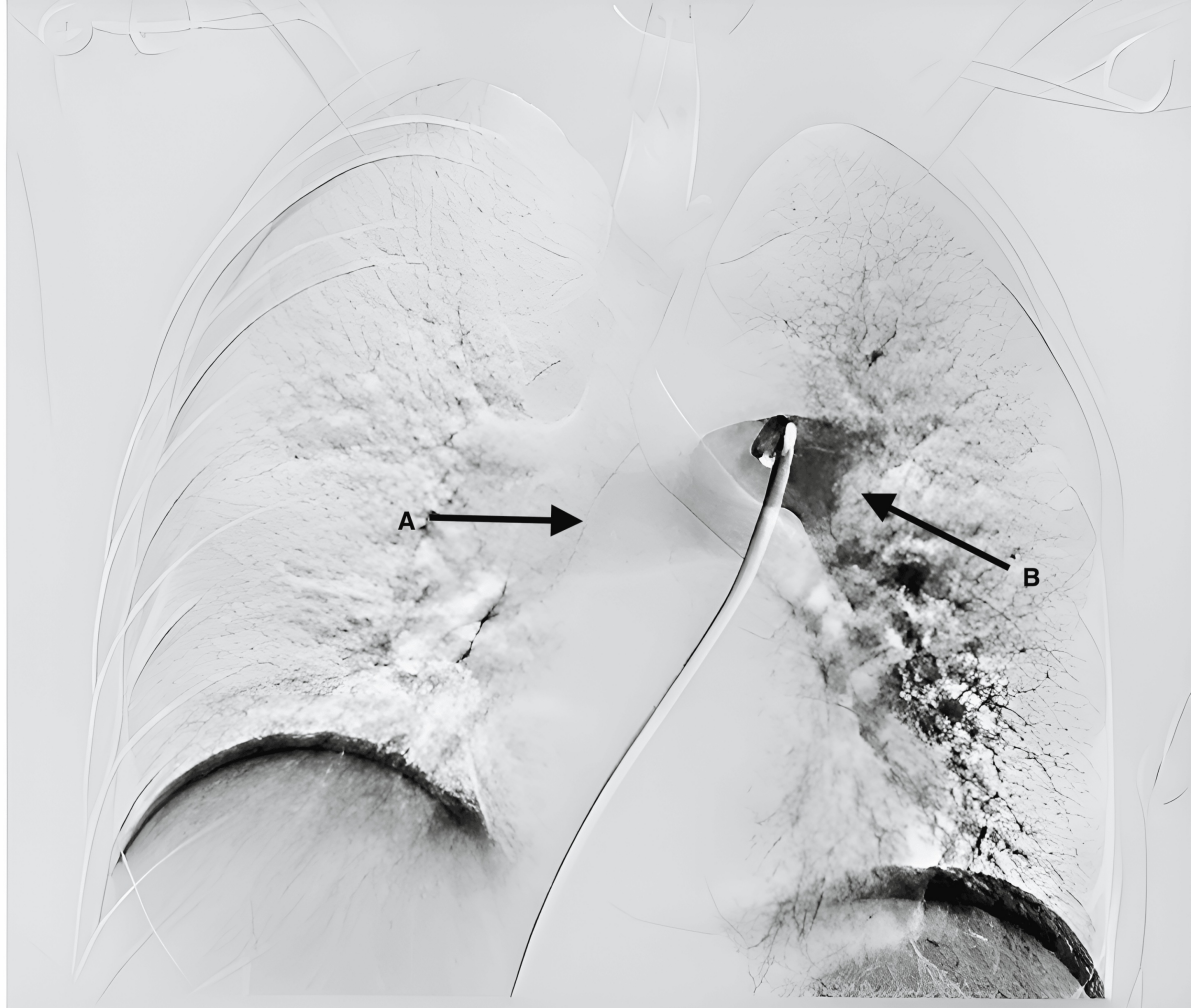
Initial pulmonary angiogram demonstrating right main pulmonary artery thrombus (A) without opacification of the right lung in addition to patent left main pulmonary artery with nonocclusive thrombus in left interlobar artery (B)

**Figure 6:**
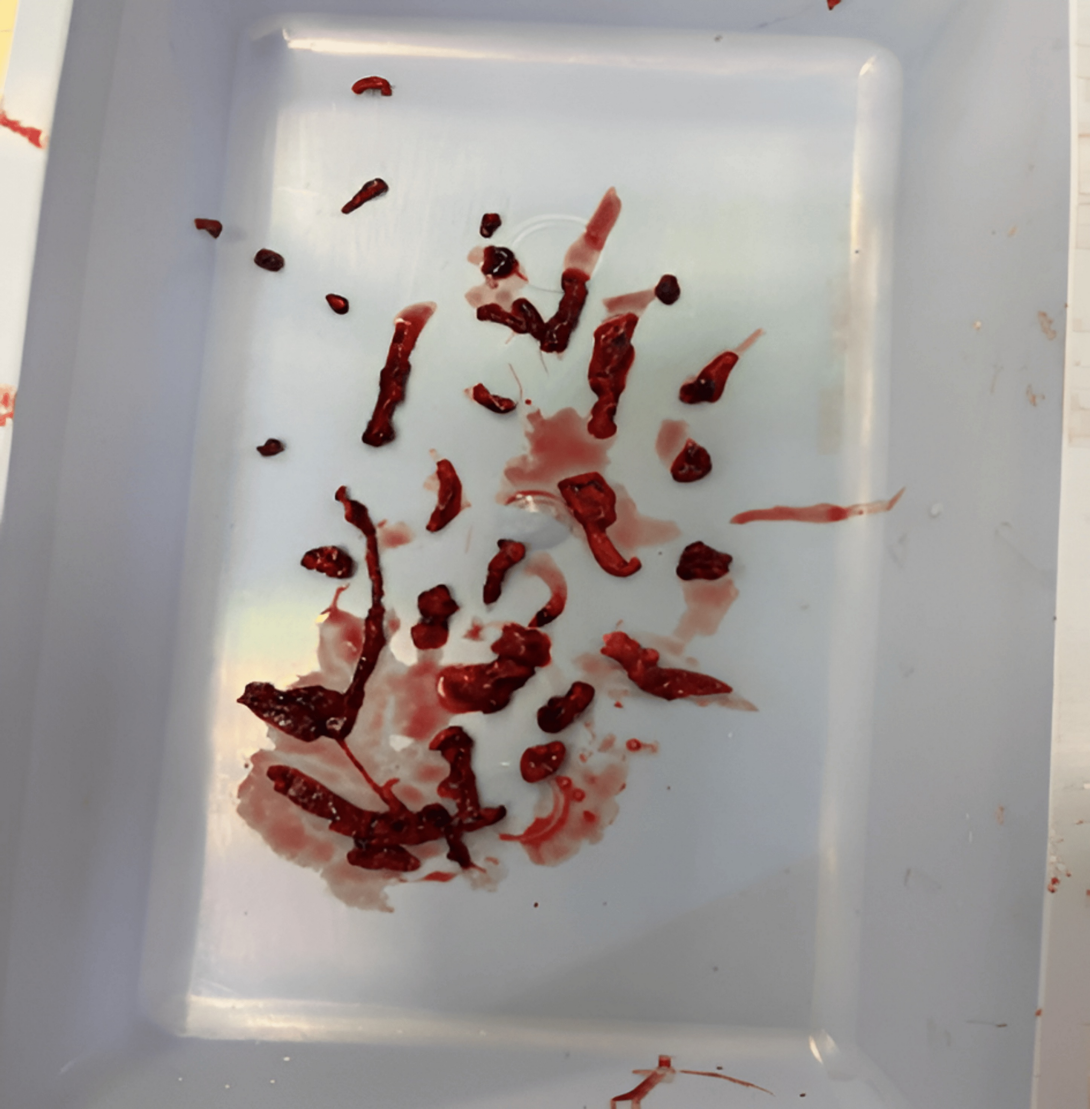
Thrombus retrieved using the Penumbra catheter

**Figure 7:**
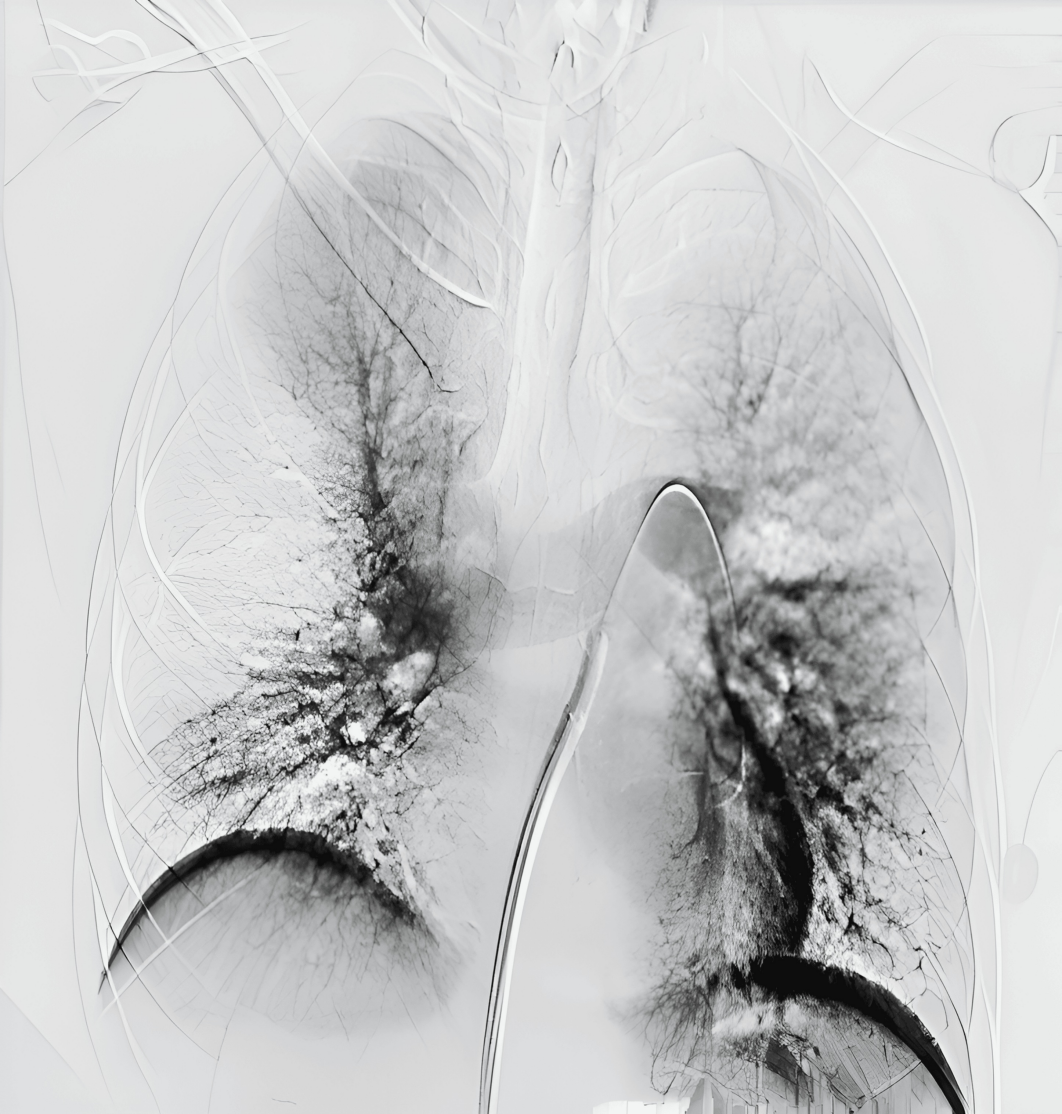
Post-thrombectomy angiogram demonstrating resolution of right and left main pulmonary artery thrombus and re-opacification of the right lung

Ultrasound imaging of the lower extremity for deep vein thrombosis showed a nonocclusive thrombus in the left popliteal and femoral veins, suggestive of an unprovoked DVT. Further imaging with computed tomography angiography (CTA) of the chest showed bilateral pulmonary embolism, more extensive on the right side without opacification of the right lung (Figure 4). An echocardiogram showed right heart strain. Clinical and imaging findings were consistent with intermediate-high-risk pulmonary embolism. Anticoagulation with intravenous heparin was initiated. In concordance with clinical guidelines, indications were met to pursue percutaneous pulmonary embolectomy. The patient underwent a pulmonary angiogram, which showed an occlusive thrombus of the main right pulmonary artery without opacification of the right lung. The left main pulmonary artery was patent with a nonocclusive thrombus (Figure 5). Thrombectomy using Penumbra Lightning CAT 12 catheter (Penumbra, Inc., Alameda, CA) was performed (Figure 6). Post-thrombectomy angiogram showed opacification of both lungs (Figure 7). Postoperatively, the patient was found to be hemodynamically stable with oxygen saturation of 100% on room air. An echocardiogram obtained showed improvement in right ventricle size and function. Prior to discharge, intravenous therapeutic heparin was bridged to warfarin with an INR goal of 2-3. The patient was discharged home at his baseline functional status and continued to do well and remained asymptomatic when seen in the outpatient clinic two months post-discharge.

## Discussion

Managing acute pulmonary embolism (PE) requires a careful consideration of treatment options to balance efficacy and safety. Thrombolysis, utilizing fibrinolytic drugs to dissolve blood clots rapidly, aims to restore blood flow in the lungs and alleviate symptoms [5]. However, its use is restricted to high-risk patients due to the associated risk of bleeding complications. Anticoagulation therapy remains the cornerstone of treatment for low-risk PE, preventing further clot formation and facilitating natural clot dissolution processes [6]. Novel interventions such as catheter-directed thrombolysis and mechanical circulatory support offer additional therapeutic avenues for high-risk patients, contributing to improved outcomes and reduced mortality rates [7]. It is important to note that, as shown in our first case, comorbidities such as diabetes, hypertension, and hyperlipidemia have not been linked to major bleeding risks, especially intracranial hemorrhage (ICH), which is a significant concern with anticoagulation and thrombolytic treatments. Recent research using German data also supports this, showing that these conditions do not increase the risk of ICH in pulmonary embolism patients treated with mechanical thrombectomy. However, the selection of treatment modality depends on individual patient factors and the severity of the condition, with a focus on minimizing adverse events and optimizing long-term prognosis [8-10].

Percutaneous pulmonary thromboembolectomy offers effective and rapid treatment for acute pulmonary embolism (PE), a condition associated with significant mortality. Surgical pulmonary embolectomy has been shown to be safe and effective, with high survival rates attributed to improved surgical technique and careful patient selection [11]. Percutaneous pulmonary embolectomy, including clot fragmentation techniques, is another option, especially for patients who might not be suitable candidates for surgery [12]. The study by QiMin et al. has demonstrated favorable outcomes in intermediate- and high-risk PEs with pulmonary embolectomy as the first-line treatment. This is evidenced by a significant reduction in mortality rates, with overall rates dropping to as low as 2.56% when excluding patients who initially received systemic thrombolysis. Additionally, patients showed marked improvement in right ventricular function during follow-ups, and no cases of recurrent PE or chronic pulmonary hypertension were reported [13]. A study by Rosovsky et al. discusses the growing use of multidisciplinary response teams to address the significant morbidity and mortality associated with pulmonary embolism (PE) in the United States. These teams are designed to rapidly convene and determine the optimal treatment approach for patients presenting with acute PE. Despite the innovation of these teams and the availability of advanced therapeutic strategies, the survival rates for high-risk PE cases remain poor. The review outlines the purpose, structure, and preliminary efficacy of these teams, emphasizing the need for further research to establish them as a new standard of care and assess their impact on patient outcomes and cost-effectiveness [14].

Catheter-directed treatments (CDT), such as thrombolysis and mechanical thrombectomy, are now increasingly used to manage acute pulmonary embolism (PE), particularly for high-risk cases. In the United States, while Europe has been slower to adopt these methods, recent changes in reimbursement policies are expected to accelerate their use. This shift could significantly increase healthcare costs associated with PE treatment. Ongoing research will determine the clinical benefits and cost-effectiveness of CDT, potentially leading to its broader integration into PE management protocols [15].

## Conclusions

This case series demonstrates the effectiveness of mechanical thrombectomy in treating acute pulmonary embolism (PE), showing significant improvements in patient outcomes. The successful interventions highlight the importance of timely diagnosis and advanced procedures in managing high-risk PE cases with excellent perioperative and short-term outcomes. Minimally invasive mechanical thrombectomy has the benefit of rapid treatment in typically high-risk, unstable patients while avoiding morbidity associated with median sternotomy, cardiopulmonary bypass, and prolonged general anesthesia. These findings support the continued use and role of mechanical thrombectomy as a vital treatment option in PE management in appropriately selected patients. Further investigations are needed in delineating its role in treating acute PEs and defining mid- and long-term outcomes.

## References

[1] De Gregorio MA, Guirola JA, Lahuerta C, Serrano C, Figueredo AL, Kuo WT (2017). Interventional radiology treatment for pulmonary embolism. World J Radiol.

[2] Jaff MR, McMurtry MS, Archer SL (2011). Management of massive and submassive pulmonary embolism, iliofemoral deep vein thrombosis, and chronic thromboembolic pulmonary hypertension: a scientific statement from the American Heart Association. Circulation.

[3] Tice C, Seigerman M, Fiorilli P, Pugliese SC, Khandhar S, Giri J, Kobayashi T (2020). Management of acute pulmonary embolism. Curr Cardiovasc Risk Rep.

[4] Lip GY, Lane DA, Lenarczyk R (2022). Integrated care for optimizing the management of stroke and associated heart disease: a position paper of the European Society of Cardiology Council on Stroke. Eur Heart J.

[5] Mohamad T, Kanaan E, Ogieuhi IJ (2024). Thrombolysis vs anticoagulation: unveiling the trade-offs in massive pulmonary embolism. Cureus.

[6] Vyas V, Sankari A, Goyal A (2024). Acute pulmonary embolism. https://www.ncbi.nlm.nih.gov/books/NBK560551/.

[7] Shah S, Ogbonna AV, Nance J (2023). A multimodality imaging approach to defining risk in patients with acute pulmonary embolism. J Am Soc Echocardiogr.

[8] Pietrasik A, Gąsecka A, Kurzyna P (2022). Characteristics and outcomes of patients consulted by a multidisciplinary pulmonary embolism response team: 5-year experience. J Clin Med.

[9] Fukuda I, Daitoku K (2017). Surgical embolectomy for acute pulmonary thromboembolism. Ann Vasc Dis.

[10] Christodoulou KC, Mohr K, Uphaus T (2024). Evolving patterns of intracranial hemorrhage in advanced therapies in patients with acute pulmonary embolism. Thromb Res.

[11] Angle FJ, Matsumoto AH, Sabri SS (2012). Percutaneous pulmonary embolectomy: Indications, techniques and outcomes. Vasc Dis Manag.

[12] Minakawa M, Fukuda I, Miyata H (2018). Outcomes of pulmonary embolectomy for acute pulmonary embolism. Circ J.

[13] QiMin W, LiangWan C, DaoZhong C (2020). Clinical outcomes of acute pulmonary embolectomy as the first-line treatment for massive and submassive pulmonary embolism: a single-centre study in China. J Cardiothorac Surg.

[14] Rosovsky R, Zhao K, Sista A, Rivera-Lebron B, Kabrhel C (2019). Pulmonary embolism response teams: purpose, evidence for efficacy, and future research directions. Res Pract Thromb Haemost.

[15] Mohr K, Keeling B, Kaier K (2024). Modelling costs of interventional pulmonary embolism treatment: implications of US trends for a European healthcare system. Eur Heart J Acute Cardiovasc Care.

